# Digital immunohistochemistry analysis of intratumoral immune infiltrates in prostate cancer patients treated with intraprostatic/systemic PSA-TRICOM vaccine

**DOI:** 10.1186/2051-1426-1-S1-P97

**Published:** 2013-11-07

**Authors:** Benedetto Farsaci, Caroline Jochems, Italia Grenga, Renee N Donahue, James L Gulley, Christopher R Heery, Ravi A Madan, Jeffrey Schlom

**Affiliations:** 1 Laboratory of Tumor Immunology and Biology, CCR, NCI, NIH, Bethesda, MD, USA

## Purpose

Preclinical studies have demonstrated that intraprostatic/intratumoral (IT) administration of vaccine in combination with systemic vaccination (SC) may improve antitumor efficacy. In this study we assessed the differential effects of IT/SC vaccination in peripheral blood mononuclear cells (PBMCs) and tumor-infiltrating lymphocytes (TILs).

## Methods

Measurements were performed before and near day 113 post IT/SC administration of PSA-TRICOM vaccine in 21 patients (pts) with locally recurrent or progressive prostate cancer (PC) enrolled in a dose-escalation phase 1 trial. Pts received initial SC vaccination with recombinant vaccinia-PSA-TRICOM and IT boosts with recombinant fowlpox (rF)-PSA-TRICOM. Cohort 3-5 received intraprostatic rF-GM-CSF. Cohort 5 received SC boosts with rF-PSA-TRICOM and rF-GM-CSF. We evaluated Treg suppression, NK function, and frequencies of immune cell subsets from PBMC by flow-cytometry. TILs were measured by digital immunohistochemistry (IHC). Prostate cores were obtained by transrectal ultrasound-guided biopsies. Prostate sections were stained for CD4, CD8, FoxP3, or isotype controls. Digital IHC images were acquired with an Aperio ScanScope AT Turbo and analyzed with the Aperio ImageScope membrane algorithm for cell membrane analysis. CD4^+^ TILs positive for FoxP3 (putative Tregs) were counted manually.

## Results

The function of Treg and NK cells isolated from PBMC, as well as circulating immune cell subsets, did not change after vaccine. A lower percentage of circulating Tregs post-vaccine correlated with decreases in serum PSA (P=0.002, R=0.67). There was an inverse correlation between CD4^+^PBMCs and CD4^+^ TILs post-vaccine (p=0.010, R=-0.61). CD4^+^ and CD8^+^ TILs increased post-vaccine (p<0.001 for both) independently of hormone status and GM-CSF treatment. Pts with low CD4^+^ TILs pre-vaccine showed a greater increase in CD4^+^ TILs post-vaccine (27 vs. 3 fold-change, respectively, p=0.002). Tregs (as % of CD4^+^ TILs) decreased post-vaccine (p<0.001). There was also a correlation between CD8^+^ TILs increase post-vaccine and decreases in serum PSA (p=0.002, R=-0.83).

## Conclusions

The use of digital IHC after IT/SC vaccination can help to document variations of immune infiltration and clinical correlates in the absence of relevant changes in PBMC immune cell subsets.

